# Prevalence of Musculoskeletal Symptoms in Patients With Hidradenitis Suppurativa and Associated Factors: Cross-Sectional Study

**DOI:** 10.2196/58989

**Published:** 2024-08-22

**Authors:** Hayley McKee, Lihi Eder, Dana Jerome, Reza D Mirza, Chikaodili Obetta, Elisabeth Pek, Vincent Piguet, Raed Alhusayen

**Affiliations:** 1 Temerty Faculty of Medicine University of Toronto Toronto, ON Canada; 2 Division of Rheumatology Department of Medicine University of Toronto Toronto, ON Canada; 3 Women’s College Research Institute Toronto, ON Canada; 4 Division of Rheumatology Sunnybrook Hospital Toronto, ON Canada; 5 Department of Health Research Methods, Evidence, and Impact McMaster University Hamilton, ON Canada; 6 Department of Medicine McMaster University Hamilton, ON Canada; 7 Division of Dermatology Department of Medicine University of Toronto Toronto, ON Canada; 8 Division of Dermatology Department of Medicine Women's College Hospital Toronto, ON Canada; 9 Sunnybrook Research Institute Toronto, ON Canada

**Keywords:** hidradenitis suppurativa, cross sectional, skin, musculoskeletal symptoms, morning stiffness, arthralgia, comorbidities, musculoskeletal, comorbidity, stiff, stiffness, muscle, muscles, muscular, prevalence, incidence, epidemiology, epidemiological, factor, factors, dermatology, dermatological

## Abstract

The prevalence of and factors associated with musculoskeletal (MSK) symptoms in patients with hidradenitis suppurativa (HS) have yet to be elucidated. Given the association between HS and inflammatory comorbidities, understanding the burden of MSK symptoms in patients with HS is crucial for patient-centered care. Our objective was to describe the prevalence of and factors associated with MSK symptoms in patients with HS. A cross-sectional study of 78 consecutive patients recruited between November 2021 and February 2023 with a dermatology-confirmed diagnosis of HS, irrespective of MSK symptoms, was performed. The average age of participants (n=78) was 37 (SD 12.2) years, and the average age at symptom onset was 23 (SD 12.1) years; 54% (n=42) of participants identified as women, and 46% (n=36) as men. The most common comorbidities included depression (n=17, 22%) and preexisting arthritis (n=12, 16%). Approximately 24% (n=18) of participants reported prolonged morning stiffness. In a multivariate regression, depression was significantly associated with morning stiffness (odds ratio [OR] 6.1, 95% CI 1.4-26.1; *P*=.02), while female sex was significantly associated with arthralgia (OR 19.1, 95% CI 1.6-235.2; *P*=.02). Every patient with depression reported arthralgia. We highlight the high prevalence of MSK symptoms among patients with HS and note the interplay between depression and MSK symptoms, with each one potentially contributing to the other.

## Introduction

Hidradenitis suppurativa (HS) is a chronic, suppurating inflammatory condition that characterizes the potential link between cutaneous and systemic disease. Currently, the underlying pathogenesis is unclear; however, follicular occlusion in the folliculopilosebaceous unit followed by follicular rupture and dysregulated immune response is widely proposed [1]. HS prevalence is 1%-4% worldwide and is higher in women [2].

Increasingly, evidence links HS, an inflammatory condition, with systemic comorbidities, such as cardiovascular disease, inflammatory bowel disease, diabetes mellitus, and depression [1]. A meta-analysis of more than 200,300 patients with HS found they had a higher prevalence of inflammatory arthritis, including spondyloarthritis and rheumatoid arthritis, than the general population [3]. Thus, this study investigated the prevalence of musculoskeletal (MSK) symptoms in patients with HS and associated factors.

## Methods

### Participants

Patients were recruited between November 2021 and February 2023 from one dermatology clinic in a tertiary hospital. All patients had dermatologist-diagnosed HS, irrespective of MSK symptoms. Of 106 patients approached, 18 declined participation and 10 withdrew; the final analysis included 78 patients. Patient information was collected via standard questionnaires, including age at first symptoms, family history, lifestyle habits, length of morning stiffness (minutes), Hurley stage, presence of arthralgia, and comorbidities including depression, diabetes mellitus, inflammatory bowel disease, hypertension, and cardiovascular disease. Rheumatic disease was investigated with follow-up X-rays, bloodwork, and rheumatology assessment.

### Statistical Analysis

Continuous variables were summarized as means, SDs, and minimums/maximums. Categorical variables were summarized using counts and percentages. Independent variables were identified a priori and examined for collinearity with the Pearson rank. Multivariable logistic regression models were built for morning stiffness (defined as stiffness and reduced mobility in the joints lasting >30 minutes after waking) and arthralgia (defined as joint pain without necessarily implying an underlying inflammatory process). Confounding was assessed by >10% change on variable removal. Odds ratios (ORs) and 95% CIs are reported in final models, with statistical significance at *P*<.05. Analyses were performed with Stata (version 17.0; StataCorp).

### Ethical Considerations

This study has been approved by the research ethics board at Sunnybrook Research Institute (1829). Informed consent was obtained from patients. Data were anonymized.

## Results

The mean age of the 78 patients with HS was 37 (SD 12.2) years at recruitment and 23 (SD 12.1) years at symptom onset; 54% (42/78) were women and 46% (36/78) were men. Most participants identified as South Asian (n=23), White (n=19), or Black (n=14). There was a family history of HS in 14% (11/78) and of rheumatic disease in 53% (41/78) of participants. The most common comorbidities included depression (n=17) and arthritis (n=12) (Table 1).

Prolonged morning stiffness was reported by 24% (18/78) of participants, while a majority of reported arthralgia (41). In a multivariate regression, depression was significantly associated with prolonged morning stiffness (OR 6.1, 95% CI 1.4-26.1; *P*=.02), while female sex was significantly associated with arthralgia (OR 19.1, 95% CI 1.6-235.2; *P*=.02). Every patient with depression reported arthralgia (Table 2).

**Table 1:** Baseline characteristics of study participants (n=78) with hidradenitis suppurativa inflammatory and musculoskeletal symptoms.

Variables					Values
**Age (years) at recruitment**					
	Mean (SD)			36.949 (12.2)	
	Range			19-67	
**Age (years) at symptom onset (n=77)**					
	Mean (SD)			23.442 (12.1)	
	Range			7-60	
**Age (years) at diagnosis**					
	Mean (SD)			29.603 (12.7)	
	Range			12-64	
**Sex, n (%)**					
	Women			42 (54)	
	Men			36 (46)	
**Ethnicity, n (%)**					
	South Asian			23 (30)	
	White			19 (24)	
	Black			14 (18)	
	Other			22 (28)	
**Employment status, n (%)**					
	Not working because of disability			10 (13)	
	Working full-time			44 (56)	
	Other			24 (31)	
**Social history, n (%)**					
	**Smoking**				
		Current smoker	16 (21)		
		Previous smoker	6 (8)		
		Never smoked	55 (71)		
	**Alcohol (n=77)**				
		Yes	26 (34)		
		No	51 (66)		
**Family history of hidradenitis suppurativa, n (%)**					
	Yes			11 (14)	
	No			67 (86)	
**Family history of rheumatic disease, n (%)**					
	Yes			41 (53)	
	No			37 (47)	
**Biologic treatment, n (%)**					
	Yes			21 (33)	
	No			43 (67)	
**Comorbidities, n (%)**					
	Arthritis			12 (16)	
	Irritable bowel disease			8 (10)	
	Diabetes mellitus			9 (12)	
	Hypertension			9 (12)	
	Cardiovascular disease			4 (5)	
	Dyslipidemia			1 (1)	
	Depression			17 (22)	
**Hidradenitis suppurativa disease severity, n (%)**					
	Hurley stage 1 or 2 (mild, moderate)			60 (79)	
	Hurley stage 3 (severe)			16 (21)	
**Musculoskeletal** **symptoms, n (%)**					
	>30 min morning stiffness			18 (24)	
	≤30 min morning stiffness			57 (76)	
	Arthralgia present			41 (84)	
	Arthralgia absent			8 (16)	

**Table 2:** Multivariate logistic regressions for participants with hidradenitis suppurativa (n=78) for symptoms of prolonged morning stiffness (>30 min) and arthralgia.

		Odds ratio (95% CI)	SE	*P* value
**Multivariate logistic regression for morning stiffness >30 min based on IBD^a^, biologic therapy, Hurley stage, depression, and age at symptom onset**				
	Depression	6.10 (1.43-26.12)	4.53	.02
	IBD	1.08 (0.13-8.86	1.16	.94
	Biologic treatment	0.71 (0.16-3.23)	0.55	.66
	Hurley stage <3	0.84 (0.17-4.09)	0.67	.83
	Age >25 years at symptom onset	0.84 (0.20-3.52)	0.62	.81
**Multivariate logistic regression for arthralgia based on IBD, sex, biologic therapy, Hurley stage, depression, and age at symptom onset^b^**				
	Female sex	19.14 (1.56-235.24)	24.51	.02
	Biologic treatment	0.30 (0.30-3.04)	0.35	.31
	Hurley stage <3	0.33 (0.17-6.60)	0.51	.47
	Age >25 years at symptom onset	6.06 (0.41-90.57)	8.37	.19

^a^IBD: inflammatory bowel disease.

^b^Depression and IBD were omitted from these rows given the perfect correlation with the predictor.

## Discussion

This cross-sectional study including 78 patients with HS demonstrates a significant association between MSK symptom severity, depression, and female sex. In a multivariate analysis, patients with depression had 6-fold greater odds of prolonged morning stiffness, while women had 19-fold greater odds of arthralgia. All patients with depression reported arthralgia.

A correlation between depression and HS has been reported [4]. The prevalence of depression in our study was 22% (17/78), comparable to 16.9% in a meta-analysis of 40,307 patients with HS [4]. World Health Organization survey data reported 69% of patients with depression present primarily with somatic symptoms, such as joint pain and back pain [5]. Proinflammatory cytokines tumor necrosis factor α (TNF-α), interleukin-1 β (IL-1β), and interleukin-10 (IL-10) are elevated in major depressive disorder [6] and HS [7], suggesting a possible pathophysiological process for increased inflammatory symptoms in comorbid patients. Alternatively, prolonged morning stiffness might limit activities of daily living, such as dressing and getting out of bed, leading to feelings of loss of independence and comorbid depression [8]. Women were more likely to report arthralgia, consistent with population-based findings of higher chronic MSK pain prevalence and inflammatory arthritis–associated pain [9] in women. Still, men may be more likely to have severe HS [10]. Further research into sex-based experiences of MSK symptoms in HS is recommended.

This cross-sectional study cannot show causality. Patients came from a single center, introducing possible selection bias. Associations between depression and arthralgia could not be assessed in the regression due to perfect correlation.

We highlight the importance of managing depression in patients with HS to lessen its potential effects on pain processing and worsened MSK symptoms. Larger cohort studies exploring the impacts of sex and depression on MSK symptoms are recommended.

## Abbreviations

HS: hidradenitis suppurativa
IBD: inflammatory bowel disease
IL-10: interleukin-10
IL-1β: interleukin-1 β
MSK: musculoskeletal
TNF-α: tumor necrosis factor α
